# Resilience Metrics in Pigs: How Novelty and Environmental Complexity Shape Affective State and Neurobiological Outcomes

**DOI:** 10.3390/ani16111742

**Published:** 2026-06-05

**Authors:** Lucas Rocha Valfré, Jessica Lucilene Cantarini Buchini, Jaqueline Murbach Braz, Yann Malini, Cristiny Santos Braga, Mateus de Andrade Da Silva, Ana Carla Moreira Cardoso, Ibiara Correia de Lima Almeida Paz, Maria Fernanda de Castro Burbarelli, Rodrigo Garófallo Garcia, Aline Cristina Sant’Anna, Luan Sousa dos Santos, Leandro Batista Costa, Fabiana Ribeiro Caldara

**Affiliations:** 1 School of Agricultural Science (FCA), Federal University of Grande Dourados (UFGD), Dourados 79824-900, MS, Brazil; jessicacantarini@gmail.com (J.L.C.B.); rodrigogarcia@ufgd.edu.br (R.G.G.); 2 Department of Animal Biosciences, University of Guelph, Guelph, ON N1G 2W1, Canada; yannmalini@yahoo.com; 3 Department of Animal Production, School of Veterinary Medicine and Animal Science (FMVZ), São Paulo State University (UNESP), Botucatu 18618-687, SP, Brazil; 4 School of Agricultural and Veterinary Sciences (FCAV), São Paulo State University (UNESP), Jaboticabal 14884-900, SP, Brazil; 5 School of Veterinary Medicine and Animal Science (FAMEZ), Federal University of Mato Grosso do Sul (UFMS), Campo Grande 79070-900, MS, Brazil; 6 Monohub–Research Group for Monogastric Animals, Graduate Program in Animal Science, School of Medicine and Life Sciences, Pontifícia Universidade Católica do Paraná (PUCPR), Curitiba 80215-901, PR, Brazil

**Keywords:** attentional bias, bioacoustics, brain-derived neurotrophic factor, cortisol, qualitative behavior assessment

## Abstract

This study evaluated whether the complexity and temporal dynamics of environmental enrichment during the nursery phase are associated with piglets’ threat-related responses and affective state using a multimodal approach. We combined an Attentional Bias Test (ABT) with bioacoustic measures, Qualitative Behavior Assessment (QBA), and biomarkers (hair cortisol and serum BDNF). In a commercial nursery setting, 675 piglets were allocated to three treatments: UNI (single continuous item: chains), ALT (chains plus an additional object rotated weekly), and SIM (continuous simultaneous provision of multiple objects). A subsample of 32 piglets underwent the ABT (UNI, *n* = 10; ALT, *n* = 11; SIM, *n* = 11). Most ABT measures related to threat-directed attention and space use showed no detectable treatment differences under the present protocol, and feeding latency was affected by stimulus exposure but not by treatment. In contrast, treatments differed in coping-related responses: UNI showed a higher frequency of excretions, ALT showed a longer standing-inactive duration, and SIM showed more escape attempts. Vocalizations were restricted to harsh grunts, with ALT showing longer harsh-grunt duration and higher total vocal output. QBA and biomarkers further differentiated treatments: SIM showed calmer/less tense expressive profiles, whereas UNI showed higher post-exposure hair cortisol and lower serum BDNF than the more complex conditions. Overall, enrichment-related differences were more consistently detected in coping-related behavior, QBA, and biomarkers than in ABT attentional measures, suggesting that enrichment implementation (stable vs. rotational) shapes partially dissociable behavioral and physiological adaptation profiles, and that both more complex strategies showed a more favorable biological profile than UNI.

## Simple Summary

After weaning, piglets must adapt to a new diet, new pen-mates, and a new environment. These changes can influence not only growth, but also how piglets cope with stress and their overall affective state. Environmental enrichment (objects pigs can explore and manipulate) is widely recommended. Yet it remains unclear whether outcomes depend on how enrichment is implemented over time (kept stable or changed regularly). Here, we compared three nursery strategies: a simple single-item option (chains), a more complex option with weekly novelty (chains plus an additional object rotated weekly), and a complex stable option with several objects available at the same time. Piglets were evaluated in an attentional bias test in which they could access a highly attractive food while exposed to an unpleasant sound, alongside behavioral measures, vocalizations, whole-animal expressive style (Qualitative Behavior Assessment), and biomarkers linked to stress and neural adaptation. Overall, the two more complex strategies produced more favorable profiles than the simple enrichment, particularly for coping-related responses and biomarkers, while attention-related outcomes in the test were less sensitive to treatment differences. These results indicate that enrichment benefits depend not only on providing objects, but also on the stable vs. rotational strategy used during the nursery phase.

## 1. Introduction

Over the past few decades, animal welfare science has shifted from an almost exclusive focus on productivity and the mere absence of suffering to a more explicit consideration of animals’ subjective experiences and the promotion of positive affective states, with the aim of enabling sustainable production and a better quality of life for animals [1]. This conceptual shift recognizes that farm animals express multidimensional affective states, commonly described in terms of valence (positive/negative) and arousal, with direct implications for physiology, cognition, and behavior [2,3,4]. Accordingly, welfare assessment should go beyond point measures of acute stress and encompass more stable patterns of reactivity, adaptation, and coping capacity over time.

This perspective is particularly relevant during the nursery phase, when weaning and social reorganization impose simultaneous nutritional, environmental, and social challenges that can alter vigilance, motivation, and exploratory repertoires. Post-weaning conditions can modulate susceptibility to persistent negative affective states, affecting attention to threats, responsiveness to change, and coping strategies [5,6,7]. In low-stimulation environments, restrictions on highly motivated behaviors such as oral activities, investigation, and exploration can increase frustration and alter animals’ responsiveness. These changes may be reflected in vocalization patterns and other signals associated with affective experience, including increased stereotypies (e.g., bar biting, non-nutritive sucking), a higher incidence of redirected behaviors (e.g., tail biting and belly nosing), apathy, reduced exploratory activity, and altered social interaction patterns [8,9,10].

This understanding has also influenced regulatory frameworks, such as Council Directive 2008/120/EC of the European Union, which establishes guidelines for pig welfare and requires permanent access to manipulable materials. In Brazil, Normative Instruction MAPA No. 113/2020 [11] adopts a similar principle, recognizing environmental enrichment as a measure to promote behavioral expression and reduce abnormal behaviors, aligning with international benchmarks and reinforcing enrichment as a structural component of housing conditions. However, responses to enrichment do not depend solely on the presence of objects. Effectiveness is shaped by properties of the stimuli and their management, such as complexity, stability, and temporal predictability. More stable environments may increase perceived safety and reduce uncertainty, whereas variation and novelty can enhance exploration and cognitive engagement [12,13]. These interactions help explain why, although widely recommended, enrichment does not always produce consistent effects on emotional and cognitive indicators; different strategies (e.g., stable/predictable versus dynamic/variable) can elicit distinct response profiles. Consequently, it is crucial to compare implementation modalities rather than simply contrasting “with” versus “without” enrichment [14,15].

To capture such changes, cognition- and emotion-based approaches have proven particularly informative. The Attentional Bias Test (ABT) enables inference about affective states by quantifying prioritization of attention toward potentially threatening stimuli when they conflict with positive stimuli such as food [3,5,16]. In pigs, this bias may manifest as increased vigilance, altered orientation responses, and longer latency to resume feeding after a threat [5,16]. In parallel, bioacoustics measures provide a noninvasive means of relating temporal and spectral vocal parameters to valence and arousal levels [10,17]. Complementarily, Qualitative Behavioral Assessment (QBA) integrates the animal’s overall expressive style and can discriminate affective states even when quantitative measures are similar [18,19,20]. Despite these advances, studies integrating ABT, QBA, and bioacoustics in piglets exposed to contrasting enrichment strategies remain scarce, particularly when the focus is on distinguishing more complex and stable environments from more dynamic and less predictable ones. The inclusion of biomarkers, such as hair cortisol (reflecting HPA axis activity over a longer time scale [21,22]) and brain-derived neurotrophic factor (BDNF, associated with neurobiological mechanisms of regulation, plasticity, and environmental adaptation [23,24,25,26,27,28]), can strengthen interpretation. Together, these approaches provide a multimodal assessment of affective state, integrating cognitive-behavioral responses, expressive behavior, and vocal expression, and allowing interpretation across different time scales, reducing reliance on any single indicator.

Against this backdrop, the aim of this study was to investigate, in an integrated manner, the effects of different environmental enrichment strategies during the nursery phase on piglets’ affective state and neurobiological, behavioral, and acoustic responses under the Attentional Bias Test (ABT). We hypothesized that higher-complexity enrichment strategies would promote a more favorable affective state and reduced reactivity to challenge compared with a low-complexity environment. Accordingly, we predicted lower cumulative physiological stress, greater neurotrophic support, calmer and less tense QBA profiles, and ABT responses indicative of improved coping capacity, such as reduced reactivity and/or faster recovery following exposure to the aversive stimulus. Specifically, we expected the UNI treatment (low complexity) to be associated with higher hair cortisol concentrations, lower BDNF levels, and more reactive or tense behavioral expressions, whereas both higher-complexity treatments (SIM and ALT) were expected to exhibit more favorable behavioral and physiological profiles. In addition, we formulated the a priori prediction that ALT (dynamic complexity with periodic novelty) would result in higher BDNF concentrations than SIM (stable complexity), based on the potential stimulatory effects of novelty on neuroplasticity-related processes. Behavioral differences between stable (SIM) and dynamic (ALT) complexity in ABT and QBA outcomes were assessed exploratorily, as no single directional prediction was established across all behavioral measures.

## 2. Materials and Methods

### 2.1. Ethical Approval

All procedures used in this study were conducted in strict accordance with national and international guidelines for animal welfare and good practices in animal experimentation. The study was approved by the Ethics Committee on Animal Use of the Federal University of Grande Dourados (CEUA/UFGD), under protocol no. 25/2025.

### 2.2. Study Location

The experiment was conducted at a commercial farrow-to-finish swine facility located in the district of Anhanduí, Campo Grande, Mato Grosso do Sul, Brazil (20°53′05.9″ S; 54°33′10.6″ W; 532 m). According to the Köppen–Geiger climate classification, the regional climate is tropical savanna (Aw) [29]. Meteorological data for the experimental period (April–May 2025) were obtained from the A702 automatic weather station in Campo Grande–MS, operated by the Brazilian National Institute of Meteorology [30]. During this period, the mean daily maximum temperature was 23.66 ± 2.95 °C, and the mean daily minimum temperature was 22.46 ± 2.64 °C. The mean daily maximum relative humidity was 73.60%, and the mean daily minimum relative humidity was 68.46%.

### 2.3. Animals and Housing

A total of 675 piglets (castrated males and females) from the commercial line TN70 (Topigs Norsvin, Curitiba, PR, Brazil) were used. Piglets were weaned 25 days of age, with a mean body weight of 6.65 ± 0.75 kg at weaning. Animals were housed in a nursery facility with raised pens with plastic slatted flooring (3.0 m × 4.5 m), equipped with automatic feeders and stainless-steel drinkers with a float valve. Feed and water were provided ad libitum throughout the experimental period. Thermal management of the facility was achieved through manual adjustments to side curtains, allowing natural ventilation to be regulated according to daily climatic conditions.

### 2.4. Experimental Design and Treatments

The experiment followed a completely randomized design, using the pen as the primary experimental unit. After standardizing the initial mean body weight across groups, treatments were randomly assigned to pens. This allocation aimed to reduce baseline variability and minimize potential confounding related to initial performance.

Fifteen pens were used (*n* = 5 pens/treatment). Each pen housed 45 piglets (0.30 m^2^/animal). Nutrition, health management, and feeding routines were standardized across pens to reduce non-experimental variability and isolate the effects of the enrichment treatments.

Treatments were defined according to enrichment complexity and temporal dynamics:

- Single Enrichment (UNI): continuous provision of branched plastic chains throughout the experimental period, representing the lowest level of environmental complexity.
- Alternated Enrichment (ALT): continuous provision of branched plastic chains plus a second device (EA1 or EA2), alternated every seven days, promoting temporal variation in the stimulus and an intermediate level of environmental complexity.
- Simultaneous Enrichment (SIM): continuous and simultaneous provision of branched plastic chains, EA1, and EA2 throughout the experimental period, representing the highest level of environmental complexity.

### 2.5. Environmental Enrichment

Environmental enrichment devices were selected based on their physical and functional properties to stimulate exploration, manipulative, and oral behaviors consistent with piglet’s natural behavioral repertoire. In all pens, objects were installed in a standardized manner regarding height and positioning, to ensure equal access and reduce location-related bias.

**Figure 1:**
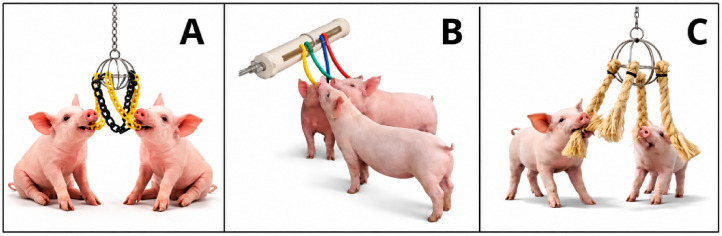
Environmental enrichment devices used in the experiment. (**A**) Branched chain; (**B**) EA1 (PVC device with hoses); (**C**) EA2 (sisal rope). Source: prepared by the authors (2026).

- Plastic Chains (CR). Chain enrichment was made of high-resistance polyethylene (black/yellow; 10 mm). Each unit consisted of six hanging chain strands (35 cm each; three yellow and three black) attached to a metal ball swivel, which provided mobility and allowed simultaneous access by multiple piglets. Chain height was defined as the distance from the pen floor to the lower end of the strands. At installation, the lower end was set at approximately 20 cm above the pen floor; thereafter, chain height was adjusted visually as piglets grew to keep the lower end within the piglets’ head region (Figure 1A).
- EA1—PVC Device. EA1 consisted of a rigid PVC tube (50 mm diameter; 90 cm length) fitted with a central metal rod. Four flexible plastic-hose sections (1/2-inch diameter; 20 cm length each) were threaded onto the rod and could slide axially along it, generating a variable mechanical response (movement/resistance) during manipulation and providing additional tactile stimulation. The device was secured to the pen side rails to ensure stability during use and suspended/fastened using a 3.2 mm steel cable (2 m per device) (Figure 1B).
- EA2—Sisal Rope. EA2 consisted of a braided natural-fiber sisal rope (14 mm diameter) configured as four hanging terminal strands (20–30 cm each) attached to the metal ball swivel, providing mobility and allowing simultaneous access by multiple piglets. The rope was suspended along the central axis of the pen. Rope height was defined as the distance from the pen floor to the lower end of the strands: at placement, the lower end was set at approximately 20 cm above the pen floor and was adjusted weekly as piglets grew to maintain a comparable relative height near the head region (Figure 1C).

#### Rotation and Cleaning Protocol

In the ALT treatment, the additional enrichment devices (EA1 and EA2) were rotated every 7 days (one cycle) as part of the dynamic novelty-based enrichment strategy. Before each reinstallation, devices were washed with neutral detergent, disinfected with 70% ethanol, and air-dried until complete evaporation. This procedure was intentionally adopted not only for hygiene purposes, but also to reduce the persistence of familiar olfactory cues associated with previously manipulated objects, thereby enhancing novelty perception and sustaining exploratory motivation over time. Sisal ropes (EA2), due to their fibrous nature and greater susceptibility to wear, were replaced with new units at the end of each cycle. In the UNI and SIM treatments, no periodic cleaning protocol was implemented in order to preserve environmental stability throughout the experimental period. In SIM, sisal ropes were replaced only when they showed clear structural wear or posed a risk of functional impairment and/or safety.

Across all treatments, devices were positioned in a standardized manner within pens, maintaining spacing between objects to avoid spatial overlap and ensuring equal access for piglets, thereby minimizing competition and location-related bias (e.g., chains and rope along the central axis; EA1 fixed to the side rails).

### 2.6. Attentional Bias Test (ABT)

A subsample of 32 piglets, randomly selected from the total of 675 animals, was used for the Attentional Bias Test (ABT) and distributed across treatments: UNI (*n* = 10), ALT (*n* = 11), and SIM (*n* = 11). Each piglet was tested once (experimental unit: one animal per test), resulting in 32 individual assessments. The testing sequence was randomized to avoid consecutive testing of piglets from the same treatment, thereby reducing potential order effects (time-related effects, assessor/animal, and fatigue). Sample size and protocol were defined based on Luo et al. [5] and Neary et al. [16].

#### 2.6.1. Arena, Context, and Behavioral Recording

**Figure 2:**
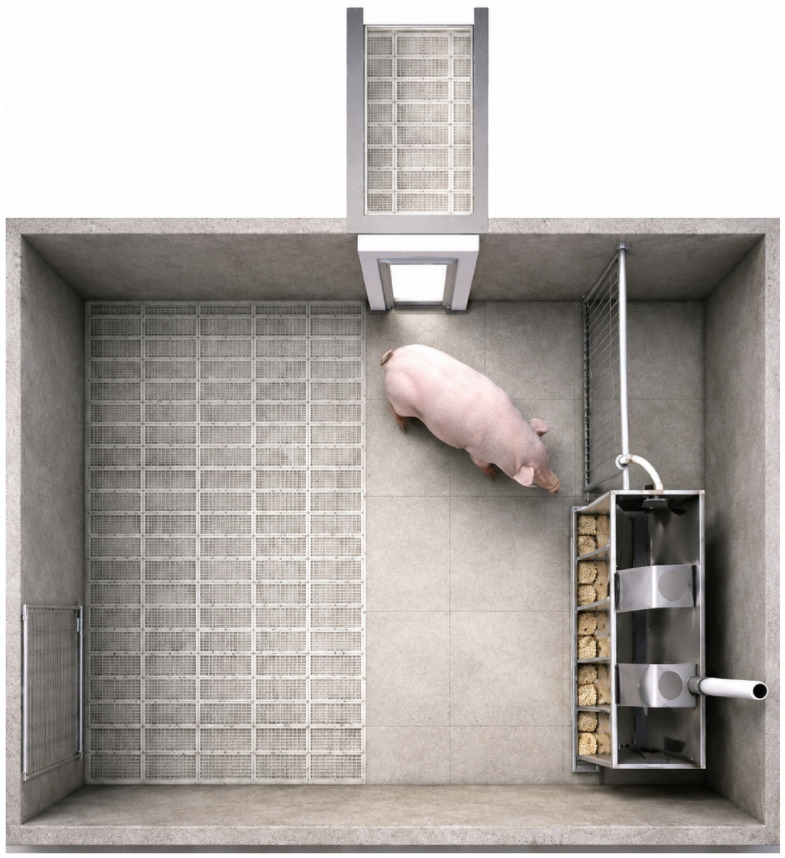
Arena for the open field test (OFT) and the novel object test (NOT), with subdivisions. Source: prepared by the authors (2026).

The ABT was conducted in a wooden arena (2.10 m × 2.10 m; 1.10 m high) with a plastic slatted floor, including a pre-arena/entry box (1.00 m × 0.70 m) separated from the test area by a guillotine door (Figure 2). To maintain consistency with the housing context and minimize responses associated with environmental novelty, the arena was set up inside a housing pen located in another nursery room. This room was physically separated from the experimental pens, thereby preserving similar flooring, lighting, and odor conditions [3,31]. This arrangement prevented experimental piglets from having prior auditory exposure to the aversive stimuli, such that sound exposure occurred only during the test session. A standard feeder was placed laterally in the arena, in a location equivalent to that used in the home pens, so that observed responses could be attributed primarily to the aversive stimulus presented during the test.

Sessions were continuously recorded using a fixed monitoring system with a high-resolution digital camera (Hikvision HD 4K, Hangzhou Hikvision Digital Technology Co., Ltd., Hangzhou, China) positioned to capture the arena from overhead (zenithal) and lateral angles, ensuring full coverage of the test. Videos were recorded in 4K resolution at 30 fps. In addition, each session was recorded individually using a smartphone (Xiaomi Redmi Note 13 Pro+ 5G; Xiaomi Corporation, Beijing, China), equipped with a 200 MP main rear camera (f/1.7, 1/1.4″ sensor, optical image stabilization—OIS). Before each test, the piglet was identified using a marker spray on a body region (head/back/flank). Immediately before the start, an identification board was shown to the camera containing date/time, treatment, replicate, and body marking, ensuring traceability and preventing duplicate testing.

#### 2.6.2. Animal Preparation and Positive Stimulus

The procedure was conducted in two stages: on day 33 of the experimental period, piglets underwent habituation to the arena and handling (5 min/animal); on day 34, the ABT was applied following a protocol adapted from Luo et al. [5] and Neary et al. [16]. Each piglet was moved individually to a holding/rest pen for 2 min before the test to reduce arousal associated with transport and to prevent prior exposure to the aversive stimulus (social contagion/observational learning).

Before each session, the feeder was supplied with pre-starter feed (a complex, attractive diet) and highly palatable food items (cucumber and sweet coconut cookies), selected based on pilot observations conducted with piglets not included in the experimental trial that showed high exploratory and feeding interest. These items were included to enhance motivation to approach the feeder and establish a feeding–attention conflict during the test. Between tests, the arena underwent mechanical cleaning to remove feces and urine, thereby reducing residual chemical cues left by the previous animal.

#### 2.6.3. ABT Protocol and Aversive Stimulus

The test was designed to assess motivational conflict between feeding (positive stimulus) and aversive auditory stimulus. Each piglet remained in the pre-arena for 10 s before being allowed access to the arena. Sessions were monitored remotely, with the observer outside the animal’s field of view to minimize interference.

The protocol used two exposures to the aversive stimulus to assess affective state and persistence of alertness. Stimuli were as follows:

- ES1 (first exposure to the aversive auditory stimulus): after 10 s of initial exploration in the arena, an audio recording of aggressive dog barking was played (10.3 s; 72 dB) through a portable loudspeaker (Eco Power EP-2315; Eco Power, São Paulo, Brazil)., positioned in a standardized location.
- ES2 (re-exposure): the same stimulus (10.3 s; 72 dB) was presented again only after the piglet had shown or effectively resumed feeding behavior following ES1, allowing assessment of recovery and maintenance of vigilance in response to the aversive cue.

While dog barking does not represent a species-specific predator cue for domestic pigs, this stimulus was selected as an abrupt, salient, and standardized auditory signal that reliably interrupts feeding behavior and increases vigilance, thereby creating a conflict between feeding motivation and attention allocation. In pigs, novel sounds may elicit startle, avoidance, and fear-related responses, depending on signal characteristics and mechanisms of novelty [32,33]. Therefore, the dog-bark recording was interpreted as an aversive auditory cue to probe attention allocation rather than as a cue to predator recognition. Accordingly, throughout the manuscript, we use “threat” as an operational term to denote this aversive auditory stimulus, without implying predator recognition.

The ABT protocol was conducted under standardized testing conditions across all sessions, with stable lighting provided by natural daylight and the farm’s standard artificial lighting system. The auditory stimulus was standardized at 72 dB using the same playback equipment and speaker positioning throughout all tests. No additional intentional auditory stimuli were introduced during testing, thereby minimizing interference from external noise. Behavioral assessment followed predefined ethogram criteria and was performed by observers with previous experience conducting Attention Bias Tests (ABT) in other species.

#### 2.6.4. Behavioral Recording and Data Collection

Data collection occurred in two stages: during the test (real time) and after the test (video analysis). In real time, immediately after the end of each aversive stimulus (ES1 and ES2), the following were recorded: (i) latency to feed (s), defined as the time interval until the piglet’s first physical contact with the food, and (ii) feeding (yes/no), coded as a binary variable. These measures were used as indicators of the stimulus effect on feeding motivation and of the persistence of alertness, inferred from feeding inhibition.

After the sessions, recordings were analyzed by a trained, blinded assessor using the Solomon Coder (Beta, DSPack version 2.3.4). Behaviors were coded using continuous sampling, with a temporal resolution of 0.2 s. State variables were quantified as duration (s), whereas event variables (e.g., escape, excretion, and vocalizations) were quantified as frequency (*n*).

The operational ethogram (Table 1) was structured by categories and included measures of arena space use, attentional bias, defensive and autonomic responses, general activity, and motivation/recovery. Threat-directed attention and freezing (defensive immobility) were defined as the main indicators of negative attentional bias. Interaction with the feeder/feeding was used as a measure of post-stimulus recovery and food motivation.

### 2.7. Bioacoustics Analysis and Emotional Inference

Bioacoustics analyses were conducted using videos recorded during the Attentional Bias Test (ABT), which simultaneously captured piglets’ behavior and vocalizations throughout each session. Vocalizations produced during the test were used as indicators of arousal and emotional valence, complementing behavioral classification with objective bioacoustics criteria. The initial identification of call types followed the criteria described by Luo et al. [5] and Neary et al. [16], with further refinement based on signal descriptors and quality parameters proposed by Diana et al. [34] and Leliveld et al. [8].

**Figure 3:**
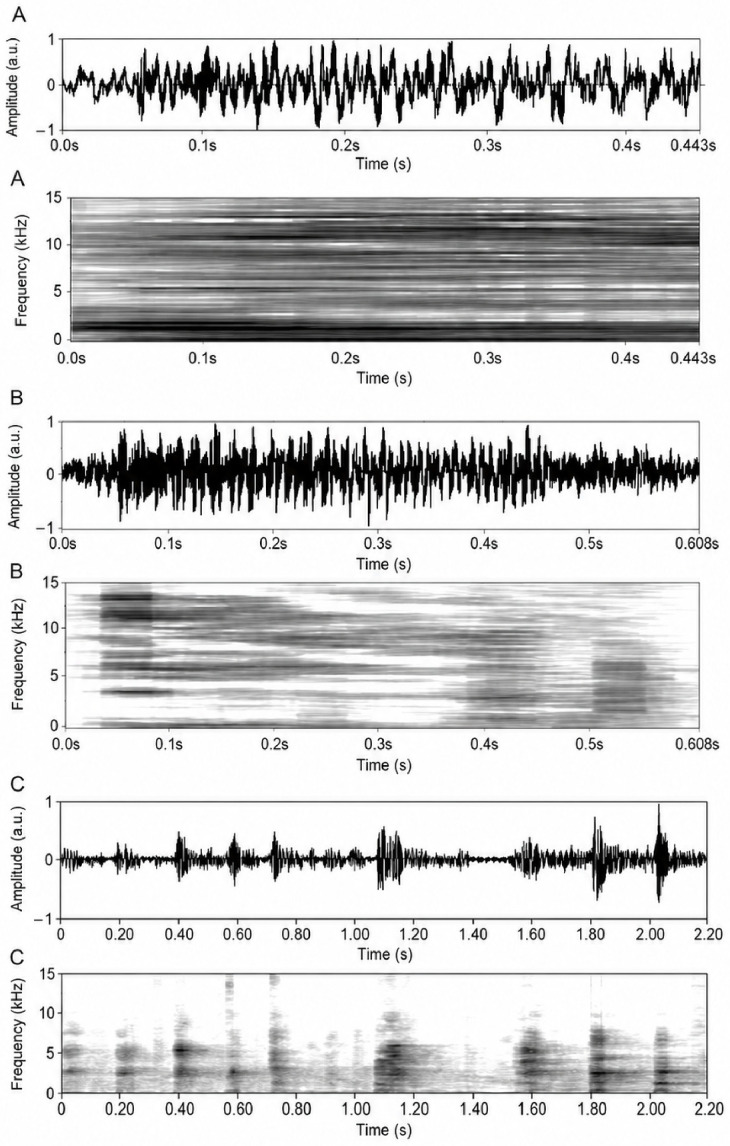
Representative spectrograms illustrating the acoustic structure of the vocal categories identified during the attentional bias test: (**A**) Bark; (**B**) Harsh grunt; and (**C**) NoseGround, a non-vocal acoustic event associated with exploratory snout contact with the substrate. Spectrograms were generated using Praat software.

Recordings were analyzed in Praat (v. 6.4.48), from which the following acoustic parameters were extracted: fundamental frequency (F0), duration, intensity, and harmonics-to-noise ratio (HNR) (Figure 3). High-frequency vocalizations were associated with states of greater reactivity/stress, reflecting negative valence and high arousal [35,36]. Barks were distinguished by an abrupt onset and a more explosive character and were therefore treated as a functionally distinct category within the vocal repertoire [37]. For low-frequency vocalizations, inference of variation in affective state was mainly based on parameters such as duration and spectral descriptors [9,10]. In addition, HNR was used as an indicator of signal quality and, by extension, as an indirect marker of emotional reactivity [8,38].

Based on these assumptions, screams and squeals were grouped into a single category (distinct from barks) because they show a convergent acoustic profile (high F0, high intensity, and low HNR) [8,10,16,35,36]. In turn, grunts were subdivided into harsh and clear grunts, based on differences in signal quality and reported associations between these patterns and states such as frustration/discomfort compared with baseline conditions [8,9,17]. Representative spectrograms illustrating the acoustic structure used to distinguish vocal categories and non-vocal acoustic events identified during signal screening are presented in Figure 3.

The integration of vocal categories and acoustic parameters resulted in an affective inference matrix, based on the two-dimensional valence–arousal model [4]. This approach enabled interpretation of variation between more tonal versus noisier signals and changes in the duration of low-frequency vocalizations across the ABT challenge. Mean values by vocal category, as well as the adopted classification criteria, are presented in Table 2.

During screening of the recordings for bioacoustics analysis, a previously uncategorized acoustic event (“NoseGround”) was identified, characterized by a sequence of broadband, atonal pulses (0–14 kHz), with no defined fundamental frequency and negative HNR (−2.20 to −3.44 dB). Because this sound was associated with exploratory behavior (snout contact/friction with the substrate) rather than a specific vocalization, and because it occurred at very low frequency, it was not included in the vocalization analyses.

### 2.8. Qualitative Behavior Assessment (QBA)

QBA was used to characterize piglets’ overall emotional expression during the ABT. The assessment was conducted for all piglets subjected to the test, based on the recorded videos (180 s per animal). Three previously trained assessors, blinded to treatments, assigned scores based on the overall impression of the animal across the entire video period, without counting isolated events [18,19]. Training included conceptual alignment, discussion of descriptors, and pilot sessions to standardize interpretation.

During the scoring phase, assessors watched the videos in the same room, while avoiding visual contact with each other and without any verbal or non-verbal communication, to ensure independence of assessments. Each video was shown in full and, immediately after playback, assessors were given 180 s to record scores. To minimize fatigue and maintain consistency throughout the session, a 15 min break was implemented after each block of five videos.

Seventeen expressive descriptors adapted for pigs based on Rutherford et al. [19] were used (Table 3). Descriptor selection was tailored to the individual, short-duration ABT context. Terms with low applicability to individual-level assessment, particularly those requiring opportunities for social interaction (e.g., sociable) or reflecting overt high-positive valence, which is more readily inferred in group/home-pen observations (e.g., content, happy), were excluded to avoid over-interpretation under constrained behavioral opportunities. In addition, descriptors with conceptual overlap (e.g., indifferent, lethargic) or that were difficult to distinguish reliably in short tests (e.g., irritable, frustrated) were removed to improve inter-assessor consistency and precision in characterizing affective states.

Each descriptor was scored using a continuous visual analog scale (0–100 mm), where 0 represented absence and 100 the maximum perceived intensity of the expressive state. Marks were subsequently measured and converted into numerical values for statistical analysis.

### 2.9. Classification of Descriptors by Emotional Valence

To standardize interpretation, the behavioral, bioacoustics, and qualitative (QBA) descriptors used in the ABT were classified a priori according to emotional valence (positive/negative) and arousal level, as summarized in Table 4. This classification supported a multimodal affective inference, in which convergence across indicators of different natures (behavioral, acoustic, and QBA) was used to strengthen interpretive consistency.

### 2.10. Biological Samples and Assays

Hair cortisol was used as an integrated indicator of hypothalamic–pituitary–adrenal (HPA) axis activity across the experimental period. Sampling was performed at two time points: on experimental day 5 (baseline/pre-exposure) and immediately after the ABT (post-exposure). The baseline estimate was obtained from 10 randomly selected piglets; these same individuals were also used for baseline blood collection for BDNF analysis, without stratification by treatment. In the post-exposure period, hair (and blood) samples were collected from all piglets treated with the ABT, and analyses were stratified by treatment.

Hair samples were obtained by clipping in the dorsal region, from the mid-dorsal area along the longitudinal midline, between the scapular region (withers apex) and the base of the lumbar spine, within an approximate area of 35 cm × 10 cm, laterally bound by about half the distance between the midline and the flanks. In all collections, inclusion of skin material was avoided. At baseline, hair was removed with scissors; post-exposure, hair was removed using a clipper, maintaining the same anatomical site for standardization.

Hair processing followed protocols described in the literature [40,41,42], and cortisol concentration was quantified by ELISA (Enzyme-Linked Immunosorbent Assay) using a commercial kit (Porcine Cortisol ELISA Kit, Elabscience^®^, Houston, TX, USA)., according to the manufacturer’s instructions. For serum analyses, blood was collected into tubes for serum separation, allowed to clot at room temperature for approximately 1 h, centrifuged (20 min at 1400× *g*, 4 °C), and serum was stored in labeled cryotubes at −80 °C until analysis. Serum BDNF concentrations were determined by ELISA (Porcine BDNF Brain Derived Neurotrophic Factor—Fine Test^®^, Wuhan, China), according to the manufacturer’s instructions.

### 2.11. Statistical Analysis

Statistical analyses of the ABT (behavioral measures and vocal/bioacoustics measures) were conducted in SAS (PROC GLIMMIX, v. 9.4). When residuals did not meet normality assumptions, generalized linear mixed models with Gamma or lognormal distributions were fitted, selecting the best-fitting model based on −2 Res Log Likelihood and AICC. Treatment comparisons were performed using adjusted means (LSMEANS), presented on the original scale via ilink, with contrasts obtained using pdiff.

Post-stimulus consumption (yes/no) was analyzed using a mixed model with binomial distribution and logit link, including treatment, stimulus (ES1 vs. ES2), and the treatment × stimulus interaction as fixed effects, and animal as a random effect to accommodate repeated measures; LSMEANS were reported as probabilities (ilink). Latency to initiate consumption was analyzed using survival methods with right censoring at 180 s: Kaplan–Meier curves (PROC LIFETEST) and between-treatment comparisons using Log-rank and Wilcoxon tests, as well as a Cox model (PROC PHREG) including treatment, stimulus, and their interaction.

For QBA, inter-rater reliability was assessed using ICC (two-way mixed-effects model, absolute agreement; ICC(3,k)). After reliability was confirmed, scores were averaged across raters and submitted to PCA (correlation matrix). Retained component scores were analyzed using linear models (OLS) with treatment as a fixed effect; in complementary analyses, sex was included as a covariate. When applicable, Tukey (HSD) was used for multiple comparisons.

Biomarkers (hair cortisol and serum BDNF) were analyzed considering that baseline corresponded to the mean obtained from 10 piglets sampled at the beginning of the experiment (distributed across treatments), which was used as a common baseline for comparisons. Treatment differences were tested based on post-ABT values (ABT animals), and additionally as variation relative to baseline (e.g., Δ = post − baseline mean), using mixed models (PROC GLIMMIX), with distributional adjustments when necessary and comparisons via LSMEANS/pdiff. In all analyses, α = 0.05 (*p* ≤ 0.05).

## 3. Results

### 3.1. Attentional Bias Test—ABT

Behaviors displayed during the ABT generally showed high within-treatment variability (large SDs) and no treatment effect for most measures of space use, attention, exploration, locomotion, food interaction, and vocalizations.

Regarding space use, there were no differences among treatments in time spent in the periphery (*p* = 0.456) or in the center (*p* = 0.366), suggesting that treatments did not detectably alter spatial distribution in the arena during the test. Similarly, indicators directly linked to the attentional component of the ABT did not differ among groups: threat-directed attention 1 (*p* = 0.647), threat-directed attention 2 (*p* = 0.479), and attention to the environment (*p* = 0.711). No differences were observed for exploration (*p* = 0.329), interaction with the feeder (*p* = 0.615), walking (*p* = 0.675), or out-of-view time (*p* = 0.877) (Table 5).

Despite this, differences emerged in responses consistent with coping strategies/reactivity: animals in the ALT group showed a longer duration of standing still (15.78 s) than those in the SIM group (5.90 s). This pattern suggests a greater component of stationary immobility in ALT, without, however, a consistent change in threat-directed attention measures. Escape attempts occurred more frequently in SIM piglets (*n* = 4.18) than in ALT piglets (*n* = 1.70) (*p* = 0.040), indicating greater expression of an active avoidance response in animals exposed to the complex and stable environment (SIM). Animals in UNI showed a higher frequency of excretion behavior (*n* = 2.00) (*p* = 0.043) than those in SIM (*n* = 1.00), suggesting greater autonomic activation in UNI under the test conditions.

There was no evidence that treatments altered the probability of consumption after aversive stimuli (*p* = 0.368), and no treatment × stimulus interaction was detected (*p* = 0.704; Table 6), indicating that the response pattern to ES1 and ES2 was statistically similar among enrichment strategies. However, a trend for a stimulus effect was observed (*p* = 0.0522), with a numerical reduction in consumption probability upon re-exposure (ES2) compared with the first exposure (ES1) across all treatments (e.g., UNI: 78.1 → 43.0%; ALT: 45.7 → 13.1%; SIM: 45.0 → 32.3%). Overall, results suggest that, under the evaluated conditions, enrichment did not detectably modify the binary decision to consume after alarm cues, although re-exposure to the stimulus was accompanied by a consistent (albeit borderline statistically) decrease in the probability of resuming consumption, compatible with greater persistence of feeding inhibition after the second stimulus.

Latency to consume was summarized by treatment using Kaplan–Meier estimates, considering as the observational unit each recorded opportunity to consume after the stimuli (i.e., 11 piglets per treatment, evaluated under two alarm sounds, totaling *n* = 22 latency observations per treatment) (Table 7).

In UNI, there was a higher proportion of episodes with consumption (19 events out of 22 observations; 13.64% censored), and central tendency estimates (median and mean) indicated generally rapid resumption of consumption after alarms in most exposures. In ALT, a lower occurrence of consumption was recorded (16/22; 27.27% censored), with a pattern compatible with longer latencies and greater heterogeneity among observations, reflected in both central tendency and the range/precision of estimates, as well as more episodes without consumption up to the 180 s limit. In SIM, despite having the same censoring proportion as ALT (16/22; 27.27%), position measures suggested that, when consumption occurred, it tended to occur earlier in some exposures, consistent with a more asymmetric distribution (some very fast events coexisting with a substantial fraction of censored observations).

However, in the Cox proportional hazards model, there was no overall treatment effect (*p* = 0.3382), and pairwise treatment comparisons were also not significant (*p* > 0.05), suggesting no detectable difference among treatments in how quickly consumption occurred. In contrast, there was a significant stimulus effect (*p* = 0.0006), indicating that feeding latencies tended to be longer after ES1 than after ES2 (faster consumption after the second exposure).

There was no treatment × stimulus interaction (*p* = 0.0831), suggesting that the stimulus effect was broadly similar across treatments. In “stimulus within treatment” contrasts, the ES1 vs. ES2 difference was marked and significant in ALT (*p* = 0.0002) and SIM (*p* = 0.0006), indicating a pronounced reduction in latency in ES2 in these groups (Table 8).

### 3.2. Bioacoustics Analysis and Inference of Affective State

During the ABT, the observed vocal repertoire was relatively restricted and dominated by low-frequency vocalizations (grunts and barks). Representative acoustic structures of the classified signals are shown in Figure 3. No screams/squeals (high-frequency vocalizations) were detected, and among grunts, only harsh grunts were observed, with no occurrence of clear grunts. Therefore, all references to “grunts” in Table 9 correspond exclusively to harsh grunts, which were interpreted as being more consistent with discomfort/frustration-related states than with calm affective states.

A treatment effect was observed for grunts (*p* = 0.0089) and, consequently, for total vocalization time (*p* = 0.0118), indicating that ALT showed greater vocal expression associated with “frustration/discomfort” than SIM, whereas UNI remained intermediate, not differing from either. Conversely, barks did not differ among treatments, suggesting that the vocal component classified as “fear/anxiety” was not detectably modulated by enrichment strategies during the ABT. Screams/squeals did not occur in any treatment, suggesting absence (or low probability, under the test conditions) of high-frequency calls often linked to high negative arousal.

However, when vocalizations were integrated according to the adopted criterion for affective-state inference, i.e., combining grunts and barks into a composite index/measure, no difference among treatments was detected. This suggests that although ALT piglets showed a greater contribution of harsh grunts, this variation did not translate into a global change in the inferred affective state derived from the set of vocalizations included in the indicator. In other words, treatments appear to have affected the form of vocal expression (the distribution among call types within the available repertoire), but not the integrated valence/arousal outcome inferred from the sum of the defined categories. This pattern is consistent with a scenario in which changes in one class (e.g., harsh grunts) are compensated by stability in another (e.g., barks), or in which individual variability and the limited repertoire (absence of clear grunts and of high-frequency calls) reduce the sensitivity of the composite indicator to discriminate treatments.

### 3.3. Qualitative Behavior Assessment (QBA)

#### 3.3.1. Inter-Rater Reliability

**Figure 4:**
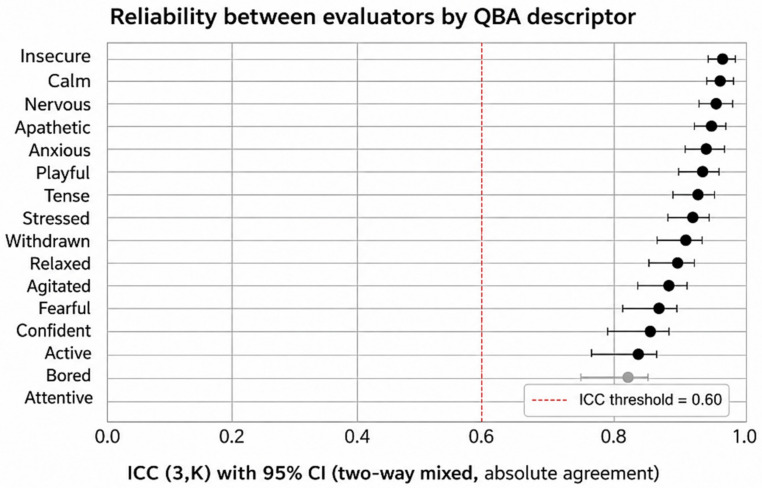
Inter-rater reliability of QBA descriptors, expressed as the intraclass correlation coefficient ICC(3,k) and respective 95% confidence intervals. The vertical dashed line represents the predefined reliability threshold (ICC = 0.60), indicating the minimum value considered acceptable for inter-observer agreement.

Inter-rater reliability for QBA descriptors was high (ICC ≥ 0.86), indicating that most score variability reflected differences among piglets rather than inconsistencies among raters (Figure 4). Therefore, mean scores across raters were used in subsequent analyses. The absence of systematic scoring bias was supported by similar patterns in descriptor means and in the overall score distribution across raters, supporting the robustness of the multivariate QBA analyses.

#### 3.3.2. Multivariate Structure of Expressive Behavior

**Figure 5:**
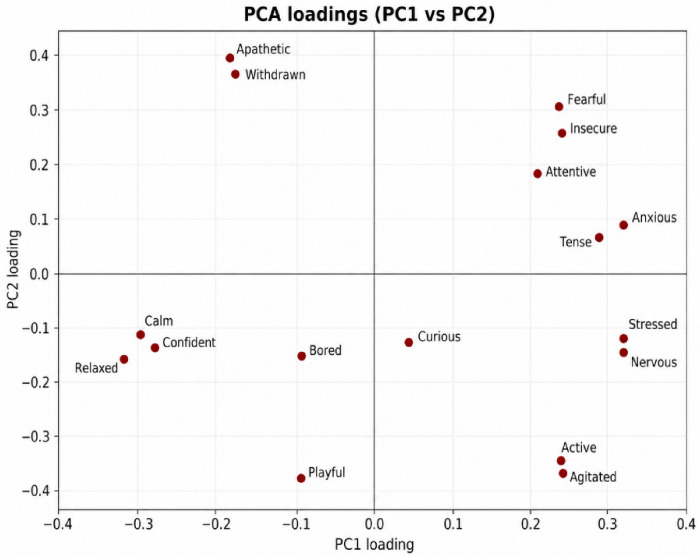
Loadings of QBA descriptors on the first two principal components (PC1 and PC2).

After score aggregation, the 17 QBA descriptors were submitted to Principal Component Analysis (PCA) based on the correlation matrix. The first two components summarized most of the variation: PC1 explained 42.09% and PC2 15.46% of the variance, totaling 57.55% (Figure 5).

**Figure 6:**
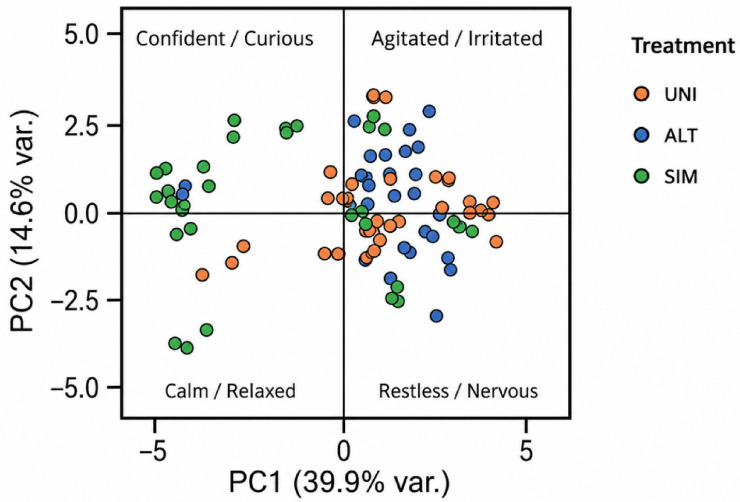
Individual piglet scores on the first two principal components (PC1 and PC2) of the QBA analysis.

PCA revealed a clear structure of expressive descriptors along the first two components (PC1 and PC2), allowing the identification of different emotional dimensions. Along PC1, descriptors such as “calm,” “confident,” and “relaxed” loaded on one side of the axis, whereas “anxious,” “tense,” “stressed,” and “nervous” loaded on the opposite side (Figure 5). This pattern indicates that PC1 primarily captured a gradient from calm/low tension to high tension/negative activation. In turn, PC2 appeared to reflect an axis related to alertness or response intensity: descriptors such as “fearful,” “insecure,” and “attentive” showed high positive loadings, suggesting increased vigilance/alertness, whereas descriptors such as “playful” and “active” loaded negatively, possibly reflecting greater positive engagement or spontaneous behavioral expression. Taken together, these results indicate that PCA captured complementary dimensions relevant to welfare assessment (valence-related expression and arousal/activation; Figure 6).

Because descriptors reflecting overt high-positive valence and social expression were not included due to the individual ABT context, PC1 should be interpreted cautiously as reflecting primarily a calm/low-tension versus high-tension gradient (i.e., “less negative” vs. “more negative”), rather than a complete high-positive valence axis.

#### 3.3.3. Treatment Effects on the Principal Components

**Figure 7:**
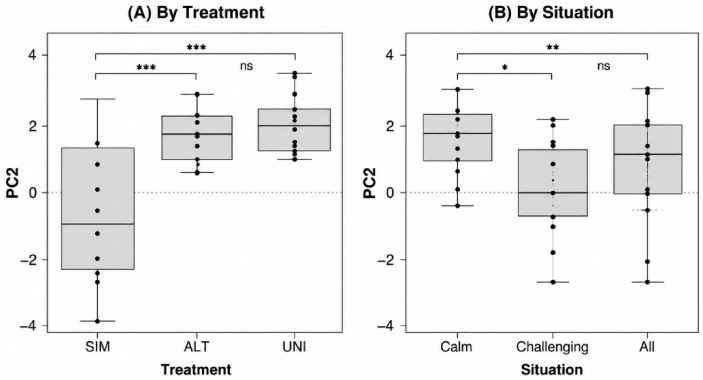
Individual piglet scores on the first two principal components (PC1 and PC2). ns = not significant; * *p* < 0.05; ** *p* < 0.01; *** *p* < 0.001.

Factor scores for PC1 and PC2 were analyzed as response variables in linear models. For PC1, an overall treatment effect was observed (F = 5.597; *p* = 0.00859; R^2^ = 0.272), and multiple comparisons indicated that SIM differed significantly from the other groups (Figure 7). Considering the interpretation of the loadings, where higher PC1 values reflect a more negative profile (fear/anxiety/tension) and lower values reflect a more positive/relaxed profile, the SIM group showed lower mean scores, suggesting a globally more positive emotional state along the main dimension synthesized by the PCA.

For PC2, there was no treatment effect (F = 0.181; *p* = 0.835; R^2^ = 0.012), with wide overlap of scores among groups (Figure 7), indicating no detectable modulation of the axis associated with behavioral activation.

### 3.4. Biomarkers of Stress and Neuroplasticity

Baseline cortisol and BDNF concentrations were tested prior to treatment allocation, and no significant differences were observed among groups, indicating homogeneity of the experimental population at the beginning of the study.

When comparing pre-exposure (common baseline, calculated from the mean of 10 piglets at the beginning of the experiment) with post-exposure, the data suggest an increase in hair cortisol in UNI and SIM, whereas ALT maintained levels with no apparent change relative to baseline. For serum BDNF, an increase was observed only in ALT, with no evidence of change in UNI and SIM (Table 10).

Considering post-exposure specifically, comparisons among treatments indicated a treatment effect for both biomarkers (Table 10). For hair cortisol, UNI showed higher values (*p* = 0.0031), while ALT and SIM did not differ from each other and remained at lower levels. For BDNF, UNI showed lower values (*p* = 0.0014), whereas ALT and SIM exhibited higher concentrations that were statistically equivalent.

## 4. Discussion

The integrated assessment of behavioral (ABT, QBA), bioacoustics, and biomarker indicators (hair cortisol, serum BDNF) suggests that environmental enrichment strategies for piglets differ not only in the presence of objects, but also in how complexity and predictability/novelty are organized over time. These differences may be reflected in distinct coping profiles and integrated physiological outcomes, aligning with the conceptual evolution of animal welfare, which incorporates affective experiences and a multidimensional interpretation of emotions [1,2,4]. In the present study, most classic ABT indicators of attentional bias (e.g., space use and threat-directed attention) did not differ across treatments, indicating no detectable effect on spatial/attentional allocation under the protocol conditions. However, significant differences emerged in defensive/autonomic responses, vocalization, and biomarkers, reinforcing the need for a multimodal framework for welfare inferences rather than relying on a single indicator [4,31,40].

The Single Enrichment Treatment (UNI), characterized by the continuous provision of a single enrichment item, resulted in a physiological profile indicative of higher cost. Post-exposure hair cortisol levels increased, exceeding those in ALT and SIM. This finding is particularly relevant because hair cortisol integrates secretion over days/weeks, reflecting the chronic dimension of stress [21,22,40,43]. In parallel, UNI showed lower post-exposure serum BDNF, consistent with reduced neurotrophic support associated with experience-induced plasticity and adjustment processes [24,27,28,44]. In the ABT, the higher frequency of excretion observed in UNI piglets, often interpreted as a marker of autonomic activation, converges with this higher-cost physiological profile and suggests lower coping efficiency in a motivational conflict context [4,45]. Taken together, these data indicate that low environmental complexity may reduce opportunities for exploratory and manipulative behaviors, thereby increasing piglets’ vulnerability to more intense physiological responses during the nursery phase, a period of high demand [7,14,15].

The Alternating Enrichment Treatment (ALT), with weekly rotation of objects, produced a more dissociated pattern. In the ABT, animals showed a longer duration of “standing still” and a lower frequency of escape attempts compared with SIM, suggesting a less active coping style along the “action” axis. From an acoustic standpoint, ALT showed longer total vocalization time, driven by harsh grunts, which is compatible with greater vocal expression associated with discomfort/frustration in the test context [8,9,10,17,35,36,39]. However, the composite affective-state index derived from vocal categories did not differ among treatments, suggesting that ALT modulated the form/temporal intensity of vocal expression without a robust shift in the aggregated outcome.

The most consistent interpretation of ALT as a challenging but not chronically costly environment comes from the neurobiological axis. ALT was the only treatment to show a detectable increase in BDNF from baseline to post-exposure, consistent with BDNF’s role in experience-dependent neural plasticity [25,27,28]. In addition, ALT showed no detectable increase in hair cortisol, indicating that enrichment rotation did not result in a higher integrated HPA-axis load [21,22,40]. A plausible hypothesis is that periodic novelty may sustain engagement and reduce habituation, but may also increase monitoring/adjustment demands, thereby producing distinct adaptation profiles [12,13].

The Simultaneous Enrichment Treatment (SIM), with the three enrichment elements continuously provided, showed a calmer/more relaxed QBA profile (PC1), consistent with the hypothesis that a complex and stable environment can promote a more positive overall expression [2,18,19,20,46]. However, SIM piglets displayed more escape attempts in the ABT, suggesting that a calmer QBA profile does not preclude a more active coping response when facing an acute challenge. This reinforces the idea that QBA and ABT may capture different dimensions: QBA reflects an overall expressive/affective style, whereas ABT emphasizes the “action” axis of coping and autonomic responses, highlighting the need to interpret domains jointly.

From a physiological standpoint, post-exposure results indicate that the main contrast occurred between UNI and the more complex conditions (ALT and SIM). ALT and SIM did not differ in hair cortisol or BDNF, whereas UNI showed higher cortisol and lower BDNF. These findings suggest that greater environmental complexity (stable or dynamic) is associated with a lower physiological stress load and a more favorable neurobiological profile. In contrast, the ABT responses appear to reflect predominant differences in coping styles (e.g., more active versus less active responses and distinct vocal-expression patterns), rather than a uniform reduction in threat reactivity across all evaluated metrics.

In summary, the results do not support a simple “ranking” among treatments, but rather combinations of characteristics that vary across domains (integrated physiology, coping under challenge, vocal expression, and qualitative assessment). This supports the notion that welfare and resilience are multidimensional phenomena that may converge in some indicators and diverge in others depending on the type of challenge and what each measure captures [4]. From an applied perspective, the findings reinforce that enrichment should be discussed beyond the binary contrast “with vs. without,” considering how it is implemented (stable vs. alternating, simultaneous diversity, and the type of resource offered), because different strategies may favor distinct dimensions of the affective state and coping style [14,15,47]. The most informative interpretation, therefore, appears from an integrated reading of indicators.

### Study Limitations

This study has some limitations that should be considered when interpreting the results. First, although the ABT sample size was defined based on previous studies using similar individual behavioral paradigms and on the logistical constraints inherent to tests conducted with one animal at a time, we acknowledge that the evaluated subsample (*n* = 32) may have limited statistical power to detect moderate or subtle treatment effects, particularly for attentional and spatial metrics, which are characterized by high inter-individual variability. Therefore, null effects observed for some ABT outcomes should be interpreted with caution and considered specific to the protocol and experimental context adopted.

Regarding biomarkers, baseline serum BDNF and hair cortisol measures were obtained prior to treatment exposure and statistically assessed for homogeneity across groups, with no significant differences detected. However, we recognize that using a common baseline does not fully eliminate the possibility of biological variability associated with pens or litters. Thus, biomarker interpretations should be considered primarily in terms of between-treatment differences at the end of the exposure period, rather than as definitive within-animal (baseline-to-post) changes attributable exclusively to enrichment management.

In addition, the generalizability of the findings may be limited to the specific nursery context and management conditions used; further studies are needed to confirm these patterns across different production systems or life stages. Finally, vocalization interpretation was intentionally cautious due to the limited vocal repertoire observed and the need to integrate bioacoustics information with other domains, avoiding isolated inferences about affective state. We recognize that the complexity of interactions among enrichment, behavior, and physiology requires broader approaches and larger samples to more fully elucidate the underlying mechanisms.

## 5. Conclusions

Overall, under the conditions of this study, environmental enrichment strategies were more clearly reflected in coping-related indicators and in neuroendocrine/neurotrophic markers than in ABT attentional measures. However, ABT results should be interpreted cautiously, as the subsample size and inter-individual variability may have limited the ability to detect differences among treatments.

From a biological perspective, the lowest-complexity environment showed the least favorable profile, being associated with a higher integrated physiological load and lower neurotrophic support. Among the more complex conditions, stable complexity was more closely associated with a calmer/more positive behavioral expression, whereas rotational novelty showed the most consistent signs of neurobiological adjustment.

Thus, the results indicate that, in commercial nursery systems, enrichment outcomes depend not only on its presence but also on how it is implemented (stable versus rotational), thereby influencing different behavioral and physiological dimensions of piglets’ adaptation.

## Figures and Tables

**Table 1:** Ethogram and behavioral coding criteria used in the Attentional Bias Test (ABT).

Category	Variable	Recording	Operational Description
Space Use	Periphery	Duration (s)	Time (s) the piglet remains in the previously demarcated peripheral zone (area adjacent to the arena walls).
	Center	Duration (s)	Time (s) the piglet remains in the previously demarcated central zone (inner arena area, excluding the periphery).
Attentional Bias	Threat-directed attention	Duration (s)	Head/gaze orientation directed toward the sound source (loudspeaker) after the end of ES1 and ES2.
	Attention to the environment	Duration (s)	Visual monitoring of the surroundings without specific orientation toward the sound source and without snout exploration, in the absence of freezing.
Defensive Responses	Freezing	Duration (s)	Sustained immobility with a rigid posture, typically with the head raised and/or ears erect, without locomotion and without active exploration.
	Escape	Frequency (*n*)	Escape attempts characterized by sudden darting/running and/or lunging against the walls/door, with an attempt to rapidly move away from the location.
	Excretion	Frequency (*n*)	Urination and/or defecation events during the session.
General Activity	Exploring	Duration (s)	Investigative behavior directed to the environment with active use of the snout (e.g., sniffing/touching the floor, walls, feeder), with or without slow locomotion.
	Walking	Duration (s)	Continuous locomotion (regular steps), not characterized as running/flight, and without snout exploration.
	Standing	Duration (s)	Standing still, without locomotion and without exploratory engagement (no sniffing/manipulating).
	Out of view	Duration (s)	Animals are located in an area that do not allow camera visualization, making it impossible to identify its posture or activity.
Motivation/ Recovery	Interaction with feeder/Consumption	Duration (s)	Contact and manipulation of the feeder/food (e.g., snout on the rim, sniffing, licking, chewing), with or without ingestion.
Vocalization	Scream/squeal	Frequency (*n*) and Duration (s)	Acute, high-intensity vocalization, typical of excitement or aversion.
	Bark	Frequency (*n*) and Duration (s)	Abrupt, short, and intense vocalization, associated with a state of alert.
	Grunt	Frequency (*n*) and Duration (s)	Low-pitched, continuous or intermittent vocalization of lower relative intensity.

Source: Adapted from Luo et al. [5] and Neary et al. [16].

**Table 2:** Acoustic parameters and criteria for categorizing pig vocalizations are used for affective inference.

Vocalization	Affective State	Peak/F0 Frequency (Hz)	Duration (S)	Intensity (Db)/HNR	Qualitative Dimension	References
Scream/Squeal	Fear, pain, anxiety	≥1000	≥0.55	≥65/<5	Noisy, atonal	[8,16,35,36]
Bark	Fear, anxiety, alertness	50–800	≤0.55	≥65/<5	Noisy, abrupt	[8,34,38]
Harsh Grunt	Frustration, discomfort	≤250	≤0.60	≤65/<5	Unstable, harsh	[8,9,10,39]
Clear Grunt	Baseline, calm	≤250	≤0.50	≤65/>15	Clear, modal	[10,17,38]

Prepared by the authors (2026). F0 = fundamental frequency; HNR = Harmonics-to-Noise Ratio. Duration and intensity values reflect the mean or the observed range for each vocalization. Scream/squeal and bark were separated based on differences in frequency, duration, and affective context, according to the literature.

**Table 3:** QBA descriptors and operational definitions used to assess piglets during the Attentional Bias Test.

Behavioral State	Operational Definition
Calm	Low reactivity, smooth movements, and stable body posture.
Relaxed	Absence of visible muscle tension and fluid behavior.
Confident	Firm posture and proactive exploration without hesitation.
Curious	Active, directed interest toward environmental stimuli.
Attentive	Balanced vigilance with a clear environmental focus.
Active	High overall level of movement and behavioral engagement.
Playful	Rapid, vigorous movements without signs of tension or aggression.
Apathetic	Low responsiveness and reduced overall activity.
Bored	Monotonous behavior with limited interaction with the environment.
Fearful	Avoidance responses, freezing, or escape attempts.
Anxious	Restless, anticipatory behavior with frequent posture changes.
Nervous	High reactivity and behavioral instability.
Tense	Rigid posture and restricted movements.
Stressed	Persistent overall expression of discomfort.
Agitated	Rapid, repetitive, or disorganized movements.
Withdrawn	Low body posture and limited exploration.
Insecure	Hesitant behavior and inconsistent responses.

Adapted from Rutherford et al. [19].

**Table 4:** Classification of descriptors according to emotional valence and arousal level.

Variable	Metric/ Indicator	Direction/Criterion	Valence	Arousal	Functional Interpretation	References
ABT						
Threat-directed attention (post-ES1 e ES2)	Duration (s)	↑ duration	Negative	High	Negative attentional bias (orientation toward threat)	[3,4,16,31]
Freezing	Duration (s)	↑ duration	Negative	High	Defensive response/hypervigilance	[4,16]
Escape	Frequency (*n*)	↑ Frequency	Negative	High	Active defensive reactivity	[4,16]
Excretion	Frequency (*n*)	↑ Frequency	Negative	High	Autonomic activation	[4]
Latency to feed	Latency (s)	↓ latency = more positive; ↑ latency = more negative	Direction-dependent	Moderate	Post-threat resilience/recovery	[3,4,16]
Time in periphery	Duration(s)	↑ duration	Negative	Moderate	Spatial avoidance	[5]
Time in center	Duration (s)	↑ duration	Neutral-to-positive	Low-to-moderate	Space use consistently with greater perceived safety	[4,5]
Exploration	Duration (s)	↑ duration	Neutral-to-positive	Low-to-moderate	Exploratory engagement/recovery (resumption)	[4,5]
Attention to environment	Duration(s)	↑ duration	Neutral-to-positive	Low-to-moderate	Non-defensive vigilance	[4,5]
Interaction with Feeder/consumption	Duration (s)	↑ duration	Positive	Low	Motivation; post-stimulus recovery	[5]
Walking/Standing (Still)	Duration (s)	↑ duration	Neutral–Contextual	Low-to-moderate	Baseline activity (interpret in context)	[3,4,5]
BIOACOUSTIC						
Scream/squeal	HNR, F0, intensity	Noisy/atonal profile (e.g., low HNR)	Negative	High	High negative arousal (fear, pain, aversion)	[8,16,35,36]
Bark	HNR + morphology	Abrupt/explosive + noisy	Negative	Moderate	Alertness; reactivity	[8,34,37]
Harsh Grunt	HNR	Low/unstable HNR	Negative	Low -to -moderate	Frustration; discomfort	[8,9,10,39]
Clear Grunt	HNR	High HNR/“modal”	Neutral-to–Positive	Low	Baseline, calmer state	[10,17,38]
QBA						
Calm; Relaxed	VAS Score	↑ score	Positive	Low	Regulation/positive low-arousal state	[18]
Confident; Curious; Attentive; Playful	VAS Score	↑ score	Positive	Moderate	Positive engagement	[19]
Fearful; Anxious; Nervous; Tense; Stressed; Agitated	VAS Score	↑ score	Negative	High	High negative arousal	[18,19]
Apathetic; Bored; Withdrawn	VAS Score	↑ score	Negative	Low	Negative low-arousal state	[4]
Insecure	VAS Score	↑ score	Negative	Moderate	Hesitation/instability	[19]
Active	VAS Score	↑ score	Neutral/Context-dependent	Variable	General activity, context-dependent	[19]

Prepared by the authors (2026). VAS—Visual Analog Scale. Note: This classification is based on the two-dimensional emotion model (valence × arousal), according to Mendl et al. [4], and each variable was categorized following the specific authors listed in the “References” column. Arrow symbols indicate the direction of the effect: ↑ increase/improvement; ↓ decrease/reduction.

**Table 5:** Means and standard errors of the mean (SEM) for the number of events (*n*) and duration (s) of different behaviors recorded in pigs during the attentional bias test.

Behavior	Treatment ^1^			SEM	*p*-Value
	UNI	ALT	SIM		
Time in periphery (s)	58.80	78.31	82.82	6.16	0.4557
Time in center (s)	121.20	101.69	97.18	6.19	0.3661
Threat-directed attention 1 (s)	6.98	5.95	7.44	1.31	0.6466
Threat-directed attention 2 (s)	1.04	0.73	2.07	0.53	0.4793
Attention to the environment (s)	13.08	24.69	21.73	3.53	0.7108
Freezing (s)	12.50	20.90	29.96	3.99	0.0562
Exploring (s)	65.58	50.56	50.45	4.23	0.3288
Interaction with feeder (s)	38.46	25.85	35.05	6.22	0.6153
Walking (s)	1.00	22.81	22.20	1.84	0.6750
Standing still (s)	8.42 ^ab^	15.78 ^a^	5.89 ^b^	1.69	0.0162
Out of view (s)	12.94	12.70	5.54	2.10	0.8769
Escape attempts (*n*)	3.10 ^ab^	1.70 ^b^	4.18 ^a^	0.59	0.0403
Excretion (*n*)	2.00 ^a^	1.50 ^ab^	1.00 ^b^	0.23	0.0432

Values with different lowercase superscript letters in the same row differ significantly at *p* < 0.05. ^1^ UNI: Single Enrichment—continuous provision of branched plastic chains throughout the experimental period; ALT: Alternating Enrichment—continuous provision of plastic chains associated with a second device (EA1 and EA2), alternated every seven days; SIM: Simultaneous Enrichment—concomitant and continuous provision of plastic chains, EA1 and EA2, throughout the experimental period.

**Table 6:** Probability of feed consumption (%) by piglets after aversive stimuli during the attentional bias test—consumption probability. Values are presented as mean ± standard error of the probabilities.

Treatment ^1^	ES1 (%)	ES2 (%)	Mean (%)
UNI	78.1 ± 16.3	43.0 ± 21.6	62.1
ALT	45.7 ± 21.7	13.1 ± 12.0	26.3
SIM	45.0 ± 22.3	32.3 ± 20.2	38.5
*p*_treat = 0.3683; *p*_stimulus = 0.052; *p*_treat × stimulus = 0.7040			

^1^ UNI: Single Enrichment—continuous provision of branched plastic chains throughout the experimental period; ALT: Alternating Enrichment—continuous provision of plastic chains associated with a second device (EA1 and EA2), alternated every seven days; SIM: Simultaneous Enrichment—concomitant and continuous provision of plastic chains, EA1 and EA2, throughout the experimental period. ES1: first threatening stimulus; ES2: second threatening stimulus.

**Table 7:** Descriptive statistics from the Kaplan–Meier analysis for feeding latency of piglets subjected to different treatments (UNI, ALT, and SIM), including number of events, censored observations, and time-to-consumption estimates.

Treatment ^1^	*n*	Events	Censored (%)	Median (s)	95% CI	Mean (s) ± SE
UNI	22	19	13.64	22.5	4–60	35.68 ± 7.80
ALT	22	16	27.27	31	0–149	60.09 ± 14.27
SIM	22	16	27.27	9.5	0–83	34.14 ± 8.10

^1^ UNI: Single Enrichment—continuous provision of branched plastic chains throughout the experimental period; ALT: Alternating Enrichment—continuous provision of plastic chains associated with a second device (EA1 and EA2), alternated every seven days; SIM: Simultaneous Enrichment—concomitant and continuous provision of plastic chains, EA1 and EA2, throughout the experimental period. *n* = number of latency observations; events = number of observations in which consumption occurred within the evaluation period; censored = observations in which the animal did not consume by the time limit (180 s). Median = time at which 50% of observations had consumed. CI = 95% confidence interval. SE = standard error.

**Table 8:** Effects of treatment, aversive stimulus (ES1 and ES2), and their interaction on piglet feeding latency, evaluated using the Cox proportional hazards model.

Effect	Comparison	β (Estimate)	SE	Wald χ^3^	*p*-Value
Treatment ^1^	—	—	—	2.17	0.3382
	UNI vs. SIM	−0.54	0.43	1.58	0.2100
	ALT vs. SIM	0.02	0.43	0	0.9692
	UNI vs. ALT	−0.56	0.43	1.69	0.1944
Stimulus ^2^	—	—	—	11.79	0.0006
	ES1 vs. ES2	−1.94	0.57	11.79	0.0006
TREAT × Stimulus	—	—	—	4.98	0.0831
	UNI vs. ALT | ES1	0.87	0.57	2.3	0.1295
	UNI vs. SIM | ES1	0.73	0.57	1.64	0.2014
	ALT vs. SIM | ES1	−0.14	0.63	0.05	0.8287
	UNI vs. ALT | ES2	−0.56	0.43	1.69	0.1944
	UNI vs. SIM | ES2	−0.54	0.43	1.58	0.2082
	ALT vs. SIM | ES2	0.02	0.43	0	0.9692
Stimulus within TREAT	ES1 vs. ES2 | UNI	−0.67	0.48	1.96	0.1610
	ES1 vs. ES2 | ALT	−2.1	0.57	13.69	0.0002
	ES1 vs. ES2 | SIM	−1.94	0.57	11.79	0.0006

^1^ UNI: Single Enrichment—continuous provision of branched plastic chains throughout the experimental period; ALT: Alternating Enrichment—continuous provision of plastic chains associated with a second device (EA1 and EA2), alternated every seven days; SIM: Simultaneous Enrichment—concomitant and continuous provision of plastic chains, EA1 and EA2, throughout the experimental period. ^2^ ES1: first threatening stimulus; ES2: second threatening stimulus. Wald χ^3^ = Wald test statistic; β = estimated regression coefficient; SE = standard error. Cox proportional hazards model with right censoring; event defined as feed consumption; observations with latency = 180 s were considered censored. *p* ≤ 0.05 was considered significant.

**Table 9:** Bioacoustics parameters and affective-state indicators of piglets subjected to different types of environmental enrichment.

Variable	Treatment ^1^			SEM	*p*-Value
	UNI	ALT	SIM		
	Vocalization				
Scream (*n*)	0.00	0.00	0.00	0.00	1.0000
Bark (*n*)	16.40	22.45	12.18	2.16	0.1162
Grunts (*n*)	11.60	19.54	6.63	2.30	0.2025
Scream/Squeal (s)	0.00	0.00	0.00	0.00	1.0000
Bark (s)	6.51	9.39	5.48	0.94	0.2249
Grunts (s)	8.67	14.32	5.53	1.77	0.3960
Total vocalization time (s)	15.16 ^ab^	23.71 ^a^	11.01 ^b^	4.80	0.0118
Affective state					
Fear/Anxiety (*n*)	16.40	22.45	12.18	2.16	0.1162
Frustration (*n*)	11.60	19.54	6.63	2.30	0.2025
Baseline/calm (*n*)	0.00	0.00	0.00	0.00	1.0000
Fear/Anxiety (s)	6.51	9.39	5.48	0.94	0.2249
Frustration (s)	8.67	14.32	5.53	1.77	0.3960
Baseline/calm (s)	0.00	0.00	0.00	0.00	1.0000

Values with different lowercase superscript letters in the same row differ significantly at *p* < 0.05. ^1^ UNI: Single Enrichment—continuous provision of branched plastic chains throughout the experimental period; ALT: Alternating Enrichment—continuous provision of plastic chains associated with a second device (EA1 and EA2), alternated every seven days; SIM: Simultaneous Enrichment—concomitant and continuous provision of plastic chains, EA1 and EA2, throughout the experimental period.

**Table 10:** Comparison between the pre-exposure period (common baseline) and the post-exposure period for serum BDNF levels and hair cortisol concentration (ng/mL), by treatment (UNI, ALT, and SIM).

Variable	Treatment ^1^		*p*-Value
	Baseline (Common)	UNI (Post-Exposure)	
Cortisol	103.66 ± 12.59 ^b^	143.21 ± 34.72 ^aX^	<0.0001
BDNF	34.76 ± 1.94 ^a^	33.40 ± 1.42 ^aZ^	0.1465
	Treatment		***p*-Value**
	Baseline (common)	ALT (post-exposure)	
Cortisol	103.66 ± 12.59 ^a^	116.70 ± 28.04 ^aY^	0.9363
BDNF	34.76 ± 1.94 ^b^	36.45 ± 2.46 ^aW^	0.0062
	Treatment		***p*-Value**
	Baseline (common)	SIM (post-exposure)	
Cortisol	103.66 ± 12.59 ^b^	123.24 ± 23.99 ^aY^	0.0056
BDNF	34.76 ± 1.94 ^a^	34.03 ± 2.29 ^aW^	0.6282

^1^ UNI: Single Enrichment—continuous provision of branched plastic chains throughout the experimental period; ALT: Alternating Enrichment—continuous provision of plastic chains associated with a second device (EA1 and EA2), alternated every seven days; SIM: Simultaneous Enrichment—concomitant and continuous provision of plastic chains, EA1 and EA2, throughout the experimental period. Values are presented as mean ± SD. Different lowercase letters within the same row (for each treatment) indicate a difference between baseline vs. post-exposure (*p* ≤ 0.05). Different uppercase letters in the post-exposure column indicate differences among treatments, with X and Y used for cortisol comparisons (*p* = 0.0031) and Z and W for BDNF comparisons (*p* = 0.0014).

## Data Availability

The data presented in this study are available upon reasonable request from the corresponding authors.

## Abbreviations

The following abbreviations are used in this manuscript:

ABT	Attentional Bias Test
ALT	Alternated Enrichment
Aw	Tropical savanna climate
BDNF	Brain-Derived Neurotrophic Factor
CEUA/UFGD	Ethics Committee on Animal Use of the Federal University of Grande Dourado’s
CR	Plastic Chains
EA1 E	Environmental Enrichment Device 1
EA2	Environmental Enrichment Device 2
ES1	First threatening stimulus
ES2	Second threatening stimulus
EU	European Union
F0	Fundamental frequency
HNR	Harmonics-to-Noise Ratio
HPA	Hypothalamic–Pituitary–Adrenal
ICC	Intraclass Correlation Coefficient
IN 113	Normative Instruction MAPA No. 113/2020
INMET	Brazilian National Institute of Meteorology
MAPA	Ministry of Agriculture, Livestock and Food Supply
OIS	Optical Image Stabilization
PCA	Principal Component Analysis
PVC	Polyvinyl Chloride
QBA	Qualitative Behavioral Assessment
SIM	Simultaneous Enrichment
UNI	Single Enrichment
VAS	Visual Analog Scale

## References

[1] Mellor D. (2016). Updating Animal Welfare Thinking: Moving beyond the “Five Freedoms” towards “A Life Worth Living”. Animals.

[2] Rault J.-L., Bateson M., Boissy A., Forkman B., Grinde B., Gygax L., Harfeld J.L., Hintze S., Keeling L.J., Kostal L. (2025). A consensus on the definition of positive animal welfare. Biol. Lett..

[3] Browning H., Veit W. (2022). The sentience shift in animal research. New Bioeth..

[4] Mendl M., Burman O.H.P., Paul E.S. (2010). An Integrative and Functional Framework for the Study of Animal Emotion and Mood. Proc. R. Soc. B.

[5] Luo L., Reimert I., De Haas E.N., Kemp B., Bolhuis J.E. (2019). Effects of Early and Later Life Environmental Enrichment and Personality on Attention Bias in Pigs (*Sus scrofa domesticus*). Anim. Cogn..

[6] Luo L., Reimert I., Middelkoop A., Kemp B., Bolhuis J.E. (2020). Effects of Early and Current Environmental Enrichment on Behavior and Growth in Pigs. Front. Vet. Sci..

[7] Lucas M.E., Hemsworth L.M., Hemsworth P.H. (2024). Review: Early Life Piglet Experiences and Impacts on Immediate and Longer-Term Adaptability. Animal.

[8] Leliveld L.M.C., Düpjan S., Tuchscherer A., Puppe B. (2017). Vocal Correlates of Emotional Reactivity within and across Contexts in Domestic Pigs (Sus Scrofa). Physiol. Behav..

[9] Friel M., Kunc H.P., Griffin K., Asher L., Collins L.M. (2019). Positive and Negative Contexts Predict Duration of Pig Vocalisations. Sci. Rep..

[10] Briefer E.F., Vizier E., Gygax L., Hillmann E. (2019). Expression of Emotional Valence in Pig Closed-Mouth Grunts: Involvement of Both Source- and Filter-Related Parameters. J. Acoust. Soc. Am..

[11] Ministério da Agricultura, Pecuária e Abastecimento (MAPA) (2020). Instrução Normativa Nº 113, de 16 de Dezembro de 2020.

[12] Dejong I., Prelle I., Vandeburgwal J., Lambooij E., Korte S., Blokhuis H., Koolhaas J. (2000). Effects of Environmental Enrichment on Behavioral Responses to Novelty, Learning, and Memory, and the Circadian Rhythm in Cortisol in Growing Pigs. Physiol. Behav..

[13] Machado S.P., Caldara F.R., Foppa L., De Moura R., Gonçalves L.M.P., Garcia R.G., Nääs I.D.A., Nieto V.M.O.D.S., De Oliveira G.F. (2017). Behavior of Pigs Reared in Enriched Environment: Alternatives to Extend Pigs Attention. PLoS ONE.

[14] Godyń D., Nowicki J., Herbut P. (2019). Effects of Environmental Enrichment on Pig Welfare—A Review. Animals.

[15] Mkwanazi M.V., Ncobela C.N., Kanengoni A.T., Chimonyo M. (2019). Effects of Environmental Enrichment on Behaviour, Physiology and Performance of Pigs—A Review. Asian-Australas. J. Anim. Sci..

[16] Neary J.M., Ali A.B.A., Jacobs L. (2024). Application of an Attention Bias Test after Surgical Castration in Piglets. Livest. Sci..

[17] Briefer E.F. (2012). Vocal Expression of Emotions in Mammals: Mechanisms of Production and Evidence. J. Zool..

[18] Wemelsfelder F., Hunter E.A., Mendl M.T., Lawrence A.B. (2000). The Spontaneous Qualitative Assessment of Behavioural Expressions in Pigs: First Explorations of a Novel Methodology for Integrative Animal Welfare Measurement. Appl. Anim. Behav. Sci..

[19] Rutherford K.M.D., Donald R.D., Lawrence A.B., Wemelsfelder F. (2012). Qualitative Behavioural Assessment of Emotionality in Pigs. Appl. Anim. Behav. Sci..

[20] Czycholl I., Skovlund C.R., Forkman B. (2026). Literature Review of the Use of Qualitative Behaviour Assessment with a Fixed List of Terms. Front. Vet. Sci..

[21] Meyer J.S., Novak M.A. (2012). Minireview: Hair Cortisol: A Novel Biomarker of Hypothalamic-Pituitary-Adrenocortical Activity. Endocrinology.

[22] Heimbürge S., Kanitz E., Otten W. (2019). The Use of Hair Cortisol for the Assessment of Stress in Animals. Gen. Comp. Endocrinol..

[23] Lucki I. (1998). The Spectrum of Behaviors Influenced by Serotonin. Biol. Psychiatry.

[24] Nithianantharajah J., Hannan A.J. (2006). Enriched Environments, Experience-Dependent Plasticity and Disorders of the Nervous System. Nat. Rev. Neurosci..

[25] Castrén E., Rantamäki T. (2010). The Role of BDNF and Its Receptors in Depression and Antidepressant Drug Action: Reactivation of Developmental Plasticity. Dev. Neurobiol..

[26] Lesch K.-P., Waider J. (2012). Serotonin in the Modulation of Neural Plasticity and Networks: Implications for Neurodevelopmental Disorders. Neuron.

[27] Rault J.-L., Lawrence A.J., Ralph C.R. (2018). Brain-Derived Neurotrophic Factor in Serum as an Animal Welfare Indicator of Environmental Enrichment in Pigs. Domest. Anim. Endocrinol..

[28] Arroyo L., Valent D., Carreras R., Pato R., Sabrià J., Velarde A., Bassols A. (2020). Neurobiology of Environmental Enrichment in Pigs: Changes in Monoaminergic Neurotransmitters in Several Brain Areas and in the Hippocampal Proteome. J. Proteom..

[29] Alvares C.A., Stape J.L., Sentelhas P.C., De Moraes Gonçalves J.L., Sparovek G. (2013). Köppen’s Climate Classification Map for Brazil. Meteorol. Z..

[30] INMET (Instituto Nacional de Meteorologia) Dados Meteorológicos—Estação Automática de Campo Grande—MS (A702). https://portal.inmet.gov.br/dadoshistoricos.

[31] Mendl M., Burman O.H.P., Parker R.M.A., Paul E.S. (2009). Cognitive Bias as an Indicator of Animal Emotion and Welfare: Emerging Evidence and Underlying Mechanisms. Appl. Anim. Behav. Sci..

[32] Talling J.C., Waran N.K., Wathes C.M., Lines J.A. (1998). Sound Avoidance by Domestic Pigs Depends upon Characteristics of the Signal. Appl. Anim. Behav. Sci..

[33] Lehoczki F., Perez Fraga P., Andics A. (2024). Family Pigs’ and Dogs’ Reactions to Human Emotional Vocalizations: A Citizen Science Study. Anim. Behav..

[34] Diana A., Carpentier L., Piette D., Boyle L.A., Berckmans D., Norton T. (2019). An Ethogram of Biter and Bitten Pigs during an Ear Biting Event: First Step in the Development of a Precision Livestock Farming Tool. Appl. Anim. Behav. Sci..

[35] Illmann G., Hammerschmidt K., Špinka M., Tallet C. (2013). Calling by Domestic Piglets during Simulated Crushing and Isolation: A Signal of Need?. PLoS ONE.

[36] Ferrari S., Costa A., Guarino M. (2013). Heat Stress Assessment by Swine Related Vocalizations. Livest. Sci..

[37] Chan W.Y., Cloutier S., Newberry R.C. (2011). Barking Pigs: Differences in Acoustic Morphology Predict Juvenile Responses to Alarm Calls. Anim. Behav..

[38] Nicolaisen T.J., Bollmann K.E., Hennig-Pauka I., Fischer S.C.L. (2025). Framework for Classification of Fattening Pig Vocalizations in a Conventional Farm with High Relevance for Practical Application. Animals.

[39] Leliveld L.M.C., Düpjan S., Tuchscherer A., Puppe B. (2016). Behavioural and Physiological Measures Indicate Subtle Variations in the Emotional Valence of Young Pigs. Physiol. Behav..

[40] Wiechers D.H., Brunner S., Herbrandt S., Kemper N., Fels M. (2021). Analysis of Hair Cortisol as an Indicator of Chronic Stress in Pigs in Two Different Farrowing Systems. Front. Vet. Sci..

[41] Casal N., Manteca X., Escribano D., Cerón J.J., Fàbrega E. (2017). Effect of Environmental Enrichment and Herbal Compound Supplementation on Physiological Stress Indicators (Chromogranin A, Cortisol and Tumour Necrosis Factor-α) in Growing Pigs. Animal.

[42] Casal-Plana N., Manteca X., Dalmau A., Fàbrega E. (2017). Influence of Enrichment Material and Herbal Compounds in the Behaviour and Performance of Growing Pigs. Appl. Anim. Behav. Sci..

[43] Gupta A., Yadav U., Bansal K.N., Bishnoi M.B., Bala R., Verma N., Bhardwaj S., Kumar P., Kumar D., Yadav P.S. (2023). Hair Cortisol: A Biomarker of Chronic Stress in Animals and Its Association with Reproduction. Anim. Reprod. Update.

[44] Park H., Poo M.M. (2013). Neurotrophin Regulation of Neural Circuit Development and Function. Nat. Rev. Neurosci..

[45] Martínez-Miró S., Tecles F., Ramón M., Escribano D., Hernández F., Madrid J., Orengo J., Martínez-Subiela S., Manteca X., Cerón J.J. (2016). Causes, Consequences and Biomarkers of Stress in Swine: An Update. BMC Vet. Res..

[46] Douglas C., Bateson M., Walsh C., Bédué A., Edwards S.A. (2012). Environmental Enrichment Induces Optimistic Cognitive Biases in Pigs. Appl. Anim. Behav. Sci..

[47] Luo L., Zande L.E.V.D., Marwijk M.A.V., Knol E.F., Rodenburg T.B., Bolhuis J.E., Parois S.P. (2022). Impact of Enrichment and Repeated Mixing on Resilience in Pigs. Front. Vet. Sci..

